# A new frontier in cancer therapy: Inositol-requiring enzyme 1 alpha (IRE1α) and taxane-induced pyroptosis

**DOI:** 10.1186/s43556-025-00336-w

**Published:** 2025-10-27

**Authors:** Tong Chen, Yirong Li, Xinghua Long

**Affiliations:** Department of Laboratory Medicine, Zhongnan Hospital of Wuhan University, Wuhan, China

In a recent study published in *Cell* [1], Xu et al. proposed Inositol-requiring enzyme 1 alpha (IRE1α) as a potential therapeutic target for triple-negative breast cancer (TNBC) to improve chemotherapy efficacy without triggering inflammatory side effects. This discovery not only provides new mechanistic insights into immune evasion and therapeutic resistance in TNBC cells but also offers a novel perspective on the complex interactions between cancer cells and chemotherapy drugs.

IRE1 functions as a stress transducer and is an endoplasmic reticulum (ER)-resident type I transmembrane protein. It contains BiP-binding and misfolded protein binding domains in the ER lumen, along with kinase and endoribonuclease catalytic domains, named the RNase domain in the cytosol. The RNase activity termed as regulated IRE1-dependent decay (RIDD) is responsible for cleaving a pool of RNAs, including mRNAs, rRNAs, and miRNAs [2]. Through the PROMIX clinical trial and immunohistochemical analyses, Xu et al. observed that in TNBC patients receiving neoadjuvant docetaxel treatment, the expression of IRE1α downstream target genes was significantly inversely correlated with cytotoxic T cell signatures. Meanwhile, patients with reduced IRE1α RNase activity exhibited increased T cell infiltration, upregulated antigen presentation gene expression, and longer progression-free survival. To elucidate the underlying mechanism, Xu et al. employed a TP53-deficient “immunologically cold” mouse model mimicking the microenvironment of human TNBC. They found that either docetaxel alone or the selective IRE1α RNase inhibitor ORIN1001 showed limited efficacy, but their combination produced a potent synergistic antitumor effect. The combination therapy induced the infiltration and activation of cytotoxic T cells, conventional type 1 dendritic cells (cDC1s), and M1-like macrophages, while counteracting the immunosuppressive function of polymorphonuclear myeloid-derived suppressor cells, effectively reprogramming the tumor microenvironment. A key mechanism driving this transformation was the significant expansion and activation of cDC1s, which efficiently primed naive CD8⁺ T cells via antigen cross-presentation by the CD103⁺ dendritic cell population in draining lymph nodes. Notably, the combination therapy drove CD8⁺ T-cell differentiation into a unique phenotype characterized by both cytotoxic and exhaustion features, contrasting sharply with the primary Type I interferon (IFN) response triggered by docetaxel alone.

Taxanes, a class of chemotherapeutic agents widely used in TNBC treatment, typically trigger inflammatory death in cancer cells by inducing double-stranded RNA (dsRNA) stress, thereby releasing immunostimulatory signals [3]. The classic dsRNA-response molecule ADAR1 maintains immune homeostasis by modifying dsRNA structure through A-to-I editing, thereby evading detection by pattern recognition receptors [4]. In contrast, this study reveals that in TNBC, IRE1α suppresses the accumulation of taxane-induced dsRNA through its RIDD activity, which specifically targets and degrades mRNAs containing IRE1α recognition sites and repetitive elements. This process directly inhibits dsRNA-triggered activation of the NLRP3 inflammasome and subsequent pyroptosis, representing a key mechanism of tumor immune evasion from chemotherapy-induced immunogenic cell death. However, since this mechanism primarily functions to degrade chemotherapy-induced dsRNA, treatment with ORIN1001 alone in TNBC under conditions lacking drug stress induces only minimal dsRNA accumulation. This is insufficient to activate an immune response in "cold tumors," leaving tumor cells unresponsive to immune checkpoint inhibitor (ICI) therapy (Fig. 1a).

**Fig. 1:**
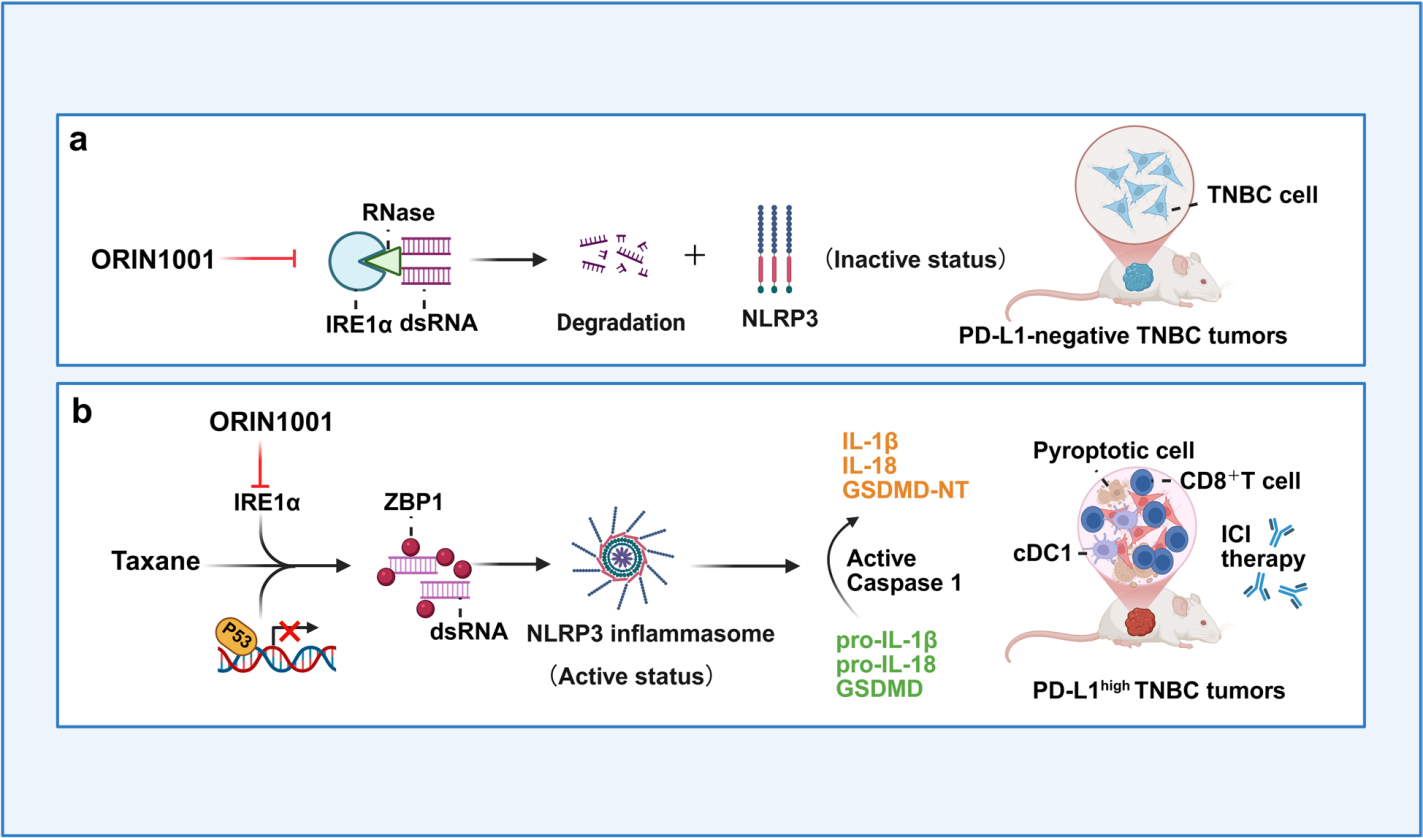
Mechanism of ORIN1001 combined with taxane to modulate TNBC immunogenicity. **a** Single-agent mechanism of action: ORIN1001 alone induces only trace dsRNA production, which fails to activate the NLRP3 inflammatory vesicles and the subsequent immune response, resulting in the persistent insensitivity of PD-L1-negative immunocold tumors to ICI treatment. **b** Mechanism of combination therapy: ORIN1001 combined with paclitaxel inhibits IRE1α function and contributes to stable dsRNA accumulation. dsRNA recognition by ZBP1 activates NLRP3 inflammatory vesicles, which induce tumor cell pyroptosis by caspase-1 cleavage of GSDMD and releases IL-1β/IL-18, accompanied by CD8⁺ T cells with cDC1 infiltration in the tumor microenvironment. Ultimately, the tumor was converted from PD-L1-negative to PD-L1 high-expression status, significantly enhancing ICI treatment sensitivity. (created in BioRender.com [https://biorender.com])

Concurrently, in TP53-mutant TNBC models treated with a combination of paclitaxel and ORIN1001, the inhibition of IRE1α-RIDD activity led to the accumulation of its target mRNAs, which subsequently formed stable dsRNA structures. These dsRNAs were specifically recognized by the cytosolic pattern recognition receptor Z-DNA binding protein 1 (ZBP1), triggering the assembly of the NLRP3 inflammasome and the activation of caspase-1. Activated caspase-1 then cleaved Gasdermin D (GSDMD), releasing its N-terminal pore-forming domain, which inserted into the plasma membrane and ultimately induced pyroptosis in cancer cells. This process was accompanied by the release of abundant inflammatory factors, such as interleukin-1β (IL-1β) and interleukin-18 (IL-18), which significantly enhanced the recruitment and activation of tumor-infiltrating lymphocytes (TILs) and CD8⁺ T cells. More critically, IFNγ secreted by the infiltrating T cells converted previously PD-L1-negative TNBC tumors into a PD-L1-high phenotype, thereby creating a favorable environment for subsequent ICI therapy (Fig. 1b).

Notably, clinical data indicate that the immunostimulatory effects of taxanes occur specifically in TNBC patients with TP53 mutations and low IRE1α RNase activity. In approximately 85% of TNBC cases, the loss of p53 protein function releases transcriptional repression on endogenous repetitive elements, leading to substantial accumulation of transcripts with the potential to form dsRNA. However, highly active IRE1α cleaves the stem-loop structures within these RNAs, preventing the formation of immunogenic stable dsRNA. Thus, p53-mediated transcriptional regulation and IRE1α-mediated post-transcriptional degradation represent a dual-layer immunosuppressive barrier that collectively maintains tumour cells in an immunologically silent state.

Although this study reveals a key mechanism of IRE1α in regulating the immunogenicity of taxanes, several limitations remain. First, the clinical sample size was limited. Future research should expand the sample size to validate the reliability of IRE1α activity as a predictive biomarker for chemotherapy efficacy. Second, the specific molecular mechanism by which ZBP1 regulates NLRP3 inflammasome activation remains unresolved and requires further exploration of the relevant signaling pathways. Additionally, as an RNase-specific inhibitor, ORIN1001 suppresses the RIDD pathway without blocking the kinase activity of IRE1α. This functional decoupling leaves the precise role of uninhibited kinase activity in ZBP1-mediated pyroptosis unclear. We hypothesize that this kinase domain may engage in complex crosstalk with the pyroptosis pathway. On one hand, the downstream JNK signaling pathway of IRE1α, as a well-established key initiator of apoptosis, may competitively antagonize GSDMD-mediated pyroptosis through sustained activation. On the other hand, it might synergize with the pyroptosis pathway to enhance immune activation by promoting the transcription of pro-inflammatory cytokines. Therefore, elucidating the independent and synergistic roles of IRE1α's two functional domains may represent a critical direction for future research.

In conclusion, the study establishes the ER stress sensor IRE1α as a key checkpoint limiting the immunogenicity of chemotherapy. Its core mechanism lies in IRE1α degrading chemotherapy-induced dsRNA via its RNase activity, which in turn suppresses pyroptosis and promotes tumor immune escape. Therefore, targeted inhibition of this function generates potent synergy with paclitaxel, effectively converting PD-L1-negative “cold” tumors into ICI-sensitive “hot” tumors. Notably, this mechanism operates independently of the classic cGAS-STING anti-tumor pathway [5]. Specifically, while the cGAS-STING pathway primarily senses cytosolic DNA to drive a Type I IFN response, the newly identified pathway utilizes dsRNA as its key ligand to trigger a more potent inflammatory response through pyroptosis. These findings suggest tumor cells may have evolved diverse and independent mechanisms to evade immune activation mediated by different nucleic acid molecules. Furthermore, given that TP53 mutations and ER stress are also prevalent in other refractory tumors, such as high-grade serous ovarian cancer and certain lung adenocarcinomas, validating the efficacy of this combination strategy in these cancers represents an important future direction. Building on this, further elucidation of IRE1α's regulatory mechanisms across a broader range of tumor types and assessment of its clinical translational potential will be crucial for developing novel anti-cancer therapeutic regimens.

## Data Availability

Not applicable.

## References

[1] Xu L, Peng F, Luo Q, Ding Y, Yuan F, Zheng L, et al. IRE1α silences dsRNA to prevent taxane-induced pyroptosis in triple-negative breast cancer. Cell. 2024;187(25):7248-7266.e7234. 10.1016/j.cell.2024.09.032. 39419025 10.1016/j.cell.2024.09.032 PMC11645245

[2] Le Goupil S, Laprade H, Aubry M, Chevet E. Exploring the IRE1 interactome: from canonical signaling functions to unexpected roles. J Biol Chem. 2024;300(4):107169. 10.1016/j.jbc.2024.107169. 38494075 10.1016/j.jbc.2024.107169 PMC11007444

[3] Tamura Y, Morikawa M, Tanabe R, Miyazono K, Koinuma D. Anti-pyroptotic function of TGF-β is suppressed by a synthetic dsRNA analogue in triple negative breast cancer cells. Mol Oncol. 2021;15(5):1289–307. 10.1002/1878-0261.12890. 33342034 10.1002/1878-0261.12890 PMC8096786

[4] Wong TL, Loh JJ, Lu S, et al. ADAR1-mediated RNA editing of SCD1 drives drug resistance and self-renewal in gastric cancer. Nat Commun. 2023;14(1):2861. 10.1038/s41467-023-38581-8. 37208334 10.1038/s41467-023-38581-8 PMC10199093

[5] Samson N, Ablasser A. The cGAS-STING pathway and cancer. Nat Cancer. 2022;3(12):1452–63. 10.1038/s43018-022-00468-w. 36510011 10.1038/s43018-022-00468-w

